# Predicting the Impact of Climate Change on Threatened Species in UK Waters

**DOI:** 10.1371/journal.pone.0054216

**Published:** 2013-01-22

**Authors:** Miranda C. Jones, Stephen R. Dye, Jose A. Fernandes, Thomas L. Frölicher, John K. Pinnegar, Rachel Warren, William W. L. Cheung

**Affiliations:** 1 School of Environmental Sciences, University of East Anglia, Norwich, Norfolk, United Kingdom; 2 Centre for Environment, Fisheries and Aquaculture Science, Lowestoft, Suffolk, United Kingdom; 3 Tyndall Centre for Climate Change Research, Norwich, Norfolk, United Kingdom; 4 Atmospheric and Oceanic Sciences Program, Princeton University, Princeton, New Jersey, United States of America; 5 Changing Ocean Research Unit, Fisheries Centre, University of British Columbia, Vancouver, British Columbia, Canada; Bangor University, United Kingdom

## Abstract

Global climate change is affecting the distribution of marine species and is thought to represent a threat to biodiversity. Previous studies project expansion of species range for some species and local extinction elsewhere under climate change. Such range shifts raise concern for species whose long-term persistence is already threatened by other human disturbances such as fishing. However, few studies have attempted to assess the effects of future climate change on threatened vertebrate marine species using a multi-model approach. There has also been a recent surge of interest in climate change impacts on protected areas. This study applies three species distribution models and two sets of climate model projections to explore the potential impacts of climate change on marine species by 2050. A set of species in the North Sea, including seven threatened and ten major commercial species were used as a case study. Changes in habitat suitability in selected candidate protected areas around the UK under future climatic scenarios were assessed for these species. Moreover, change in the degree of overlap between commercial and threatened species ranges was calculated as a proxy of the potential threat posed by overfishing through bycatch. The ensemble projections suggest northward shifts in species at an average rate of 27 km per decade, resulting in small average changes in range overlap between threatened and commercially exploited species. Furthermore, the adverse consequences of climate change on the habitat suitability of protected areas were projected to be small. Although the models show large variation in the predicted consequences of climate change, the multi-model approach helps identify the potential risk of increased exposure to human stressors of critically endangered species such as common skate (*Dipturus batis*) and angelshark (*Squatina squatina*).

## Introduction

The last 100 years have seen significant changes in the global climate that are very likely to be attributed to anthropogenic greenhouse gas emissions [1]. Mean global surface temperature has increased by approximately 0.1°C per decade since the late 1950s and is projected to be 1.4–2.1°C above pre-industrial levels by 2050 [1], with temperatures increasing in the Arctic at almost twice the global rate in the last century. Furthermore, the ocean is becoming more acidic and less oxygenated [1], [2]. Climate change has been observed to be having a profound effect on both marine and terrestrial biodiversity [3]–[5], and this trend is expected to continue, with associated changes in species compositions [6], distributions [4] and phenological patterns [7]. Concern over the impact of climate change in the marine environment is also increasing, with longer-term shifts in mean environmental conditions and climatic variability moving outside the bounds within which adaptations in marine communities have previously been associated [8]. The changes in abundances and distributions that result from these ocean-atmospheric changes may severely impact the biological and environmental functioning of ecosystems or food webs [9], the goods and services derived from them and conservation and resource management [10], [11].

The effects of climate change on threatened or endemic species (those unique to a defined geographic area) are of particular concern. These species are frequently restricted to relatively small areas and population sizes and may have highly specific habitat requirements, likely reducing their adaptive capacity to climatic change [12]. In addition, lack of knowledge or data concerning the abundance, dispersal and life history characteristics of threatened species is common. Recent years have thus seen an increase in studies attempting to assess how climate change might impact threatened and endemic species in terrestrial environments [13]–[15] and how conservation goals and actions should adapt in a changing climate [16]–[18]. There are far fewer studies, however, that attempt to assess the impacts of environmental and climate change on threatened marine vertebrate species. This is likely due to the issue of scarce and unreliable data available for the marine environment [19]. Furthermore, there has been little attempt to assess the interactions between climate change and other anthropogenic stressors, such as fishing, on threatened marine species.

Climate and ocean changes may also affect threatened species by influencing the efficacy of measures designed to protect them. Specifically, marine protected areas are a major tool to conserve marine biodiversity [20] and have been shown to enhance population resilience to climate-driven disturbance [21]. However, their effectiveness may itself be influenced by climate change. For example, future climate change has been predicted to reduce the amount of suitable habitat for particular species that falls within current protected areas [18], [22], thereby reducing its future conservation value. There is a need to increase the robustness and enhance resilience of protected areas to climate change [23], [24]. By assessing the degree of future environmental change within proposed protected areas, conservation planning may thus be used to protect against biodiversity loss [25], [26].

Species Distribution Modelling has been widely used to predict the potential impacts of climate change on both terrestrial [27]–[29] and marine species [30]–[32]. The bioclimatic envelope is defined here as a set of physical and biological conditions suitable for a given species [33] and is frequently obtained by using statistically or theoretically derived methods to associate current climatic variables with species occurrences. By predicting a species' current range as the manifestation of habitat characteristics that limit or support its existence at a particular location, a shift in that range may be elucidated by assessing shifts of the bioclimatic envelope under climate change scenarios. Species Distribution Models (SDMs) are able to predict species' distributions with presence only data and also perform well under small sample sizes (see [34]–[36] for an overview of methods). Applications of SDMs have been criticised [37] and it is acknowledged that some SDMs over-simplify the mechanisms determining species' distributions. However, recently developed modelling approaches have increasingly addressed these criticisms [38], [39]. SDMs also remain useful in exploring the possible magnitude and direction of species' distribution shift under climatic change. Furthermore, key uncertainties in using SDMs to assess climate change impacts on marine biota, which stem from the differences in the structure of the SDMs and the underlying climate forcing, can be explored by comparing outputs from multiple SDMs and climate models. Using multiple SDMs with a range of complexity, data requirement and statistical mechanisms is therefore a more robust way to assess species' distributions [40]. Climate scenarios developed from multiple models are also considered to be more robust than using a single model as climate models vary in complexity and reliability, with uncertainty being introduced by data input as well as interpolation method. There is therefore a need to compare future species' distribution predictions made using alternative SDM algorithms, Global Climate Models (GCMs) and species' occurrence/environmental tolerance data. The uncertainties in outputs resulting from these variations help us understand the range of potential predictions, the extent of agreement between them as well as possible extremes.

This study aims to assess the potential impact of climate change on a set of threatened species (under the International Union for Conservation of Nature (IUCN) Red List of Threatened species) predominantly inhabiting the North Sea, Northeast Atlantic and Mediterranean Sea. These species are primarily threatened by overfishing through being by-catch of commercially important fisheries [41]–[44]. They are vulnerable to fishing due to particular life history characteristics which make them intrinsically sensitive to overexploitation, such as large body sizes, late maturation and consequential slow rates of population increase [45],[46]. We express the level of impacts on these threatened species in terms of changes in range area, changes in habitat suitability throughout the species' ranges and within key protected areas around the UK, and of the possibility of bycatch. The latter is indicated by the predicted range overlap between threatened species and commercially exploited species. We hypothesize that the relative suitability of protected areas for threatened species would change as climate and ocean conditions change, thus influencing their efficacy in protecting threatened species. If both the threatened and targeted species respond similarly in direction and magnitude of distribution shift, the range overlap between species will remain similar under climate change. In contrast, if the response to climate change is species specific [47], [48] and varies to a large degree, change in overlap may be expected. We examine these hypotheses by using three modelling approaches, AquaMaps, Maxent and the Dynamic Bioclimate Envelope model (DBEM) [38], [40], to project future changes in distributions of threatened and commercially exploited species in the North Sea, and their changes relative to the distributions of example protected areas. We also examine uncertainty of the projections. Finally, we discuss the implications of results found on the threat facing these species, their likely persistence and on the conservation value of protected areas.

## Methods

### Modelling Approaches

We applied three Species Distribution Models to predict the distributions of seven threatened and ten targeted fish species (Table 1). The SDMs are summarized here and described in greater detail in the supplementary information (File S1) and publications indicated. Two of these, Maxent [49] and AquaMaps [19], apply a statistical approach to model species' distributions. These models were designed to overcome the problem of small sample sizes in presence-only datasets [50] and the lack of data and knowledge for many marine species respectively. Maxent [51] and AquaMaps [19] both use generative approaches to estimate the environmental co-variates conditioning species' presence from presence only occurrence data and a suite of environmental variables. Using presence only data enabled the potential use of the increasing quantity of publically available datasets and was also considered more appropriate as recorded absence at a location may not reflect true absence or may not result from tolerance limits of environmental variables included in the models. While Maxent applies a complex methodology, based on the principle of maximum entropy, AquaMaps uses simple, numerical descriptors of species' relationships with environmental variables to predict distributions from occurrence databases (see supplementary information, File S1). Species' current distributions (averaged over 30 years from 1971 to 2000) were predicted by associating species' occurrence data with averaged ‘current’ environmental data (1971–2000), thereby obtaining a bioclimatic envelope for each species. Models trained on the set of current environmental data were then ‘projected’ by applying them to the same environmental variables representing future climate.

**Table 1:** Commercially targeted and threatened species selected for the study.

Commercially targeted species		
Scientific name	Common Name	Landed Value 2010 (£ million)
*Nephrops norvegicus*	Norway lobster	95.3
*Lophius piscatorius*	Anglerfish/Monkfish	38.5
*Melanogrammus aeglefinus*	Haddock	36.2
*Gadus morhua*	Atlantic cod	28.6
*Solea solea*	Common Sole	14.0
*Pollachius virens*	Saithe	12.4
*Merluccius merluccius*	European Hake	10.2
*Lepidorhombus whiffiagonis*	Megrim	10.1
*Merlangius merlangus*	Whiting	9.4
*Microstomus kitt*	Lemon sole	6.3

Threatened species			
Scientific name	Common Name	IUCN Red List	Other lists
*Dipturus batis*	Common skate	Critically Endangered	OSPAR, BAP
*Squatina squatina*	Angelshark	Critically Endangered	OSPAR, BAP
*Raja undulata*	Undulate ray	Endangered	BAP
*Rostroraja alba*	White skate	Endangered	OSPAR, BAP
*Leucoraja circularis*	Sandy ray	Vulnerable	BAP
*Raja clavata*	Thornback ray	Near Threatened	OSPAR
*Scyliorhinus stellaris*	Nursehound	Near Threatened	

(OSPAR: Convention for the Protection of the Marine Environment of the North-East Atlantic; BAP: UK Biodiversity Action Plan.)

Expert opinion was incorporated into Maxent and AquaMaps to refine predictions by eliminating (‘clipping’) areas that were currently outside known occurrence ranges, including reported occurrence/absence in large ocean basins [delineated by the United Nations' Food and Agricultural Organisation (FAO) statistical area, www.fao.org/fishery/area/search/en] or depth limits [40]. The use of large ocean-basin and wide depth limits in ‘clipping’ considered both the current and potential future-shifted distribution. The ‘clipping’ procedure avoided over-prediction of relative habitat suitability in areas of the world where species are known not to occur, or which are unsuitable due to depth. Although depth was included in each model to retain the relative habitat suitability due to depth, maximum tolerance limits may be over-estimated in Maxent and AquaMaps due to the relatively low resolution of depth and occurrence data, in particular at the edge of the continental shelf, thereby over-predicting range extent. Maximum depth limits obtained from Fishbase [52] were increased by 50% in predictions for both time periods. This allowed for the deepening of species with ocean warming that has been observed [53] while preventing difference in predictions between the two time periods being inflated by applying different depth cut off points.

The third model, DBEM [38], combines statistical and mechanistic approaches in predicting species' distributions. It attempts to avoid the bias that might be introduced by the skewed distribution of sampling effort present in many datasets collected sporadically. Firstly, the associated *Sea Around Us Project* model [54] is used to predict a species' current distribution based on a set of ‘filters’, restricting a distribution based on known parameters, geographic limits or habitat preferences. Filters were applied for FAO area, habitat, latitudinal limits and depth. The DBEM then uses the predicted current distribution to define a species' bioclimatic envelope by its ‘preference profile’ (the relative suitability of different environmental values) for each environmental variable. Change in a species' relative abundance following changing environmental conditions is then simulated by incorporating a population growth model [33] as well as ecophysiological parameters [33], [38] (see supplementary information, File S1). Comparison between model hindcast and historical distribution changes of fishes and invertebrates from the 1970s to the 2000s in the Bering Sea and Northeast Atlantic suggest that DBEM has significant predictive skills for species distribution shifts in these regions [55].

### Species' occurrence data

Two sets of species were selected to investigate how altered range distributions under climate change might impact species that are threatened by overfishing through bycatch. We assume that the degree of range overlap between a commercially targeted species and one classified here as ‘threatened’ is an indication of bycatch potential of the threatened species. Ten commercially targeted demersal species, being the top nine fish species and the top invertebrate species by value of landings that were caught by fleets in UK waters in 2006–2010 (Marine Management Organisation, MMO) [56], were included (Table 1). Although some of these species may also be listed as endangered, for example under the IUCN Red List [41], they are still considered main commercial species by the fisheries. A further set of species of conservation concern, henceforth ‘threatened’, was chosen from the IUCN Red List of Threatened Species [41], the Convention for the Protection of the Marine Environment of the North-East Atlantic (‘OSPAR’ Convention) List of Threatened and/or Declining Species [57], and the UK Biodiversity Action Plan (UK BAP) priority marine species [58]. These species are specifically threatened by bycatch and have ranges restricted to the North Sea, East Atlantic Ocean and Mediterranean Sea (Table 1).

Species occurrence data were obtained from three global online databases: the International Council for Exploration of the Sea (ICES) EcoSystemData database (http://ecosystemdata.ices.dk); the Ocean Biogeographic Information System (OBIS) (Vanden Berghe, 2007; http://www.iobis.org) and the Global Biodiversity Information Facility (GBIF) (http://data.gbif.org), all last accessed in 2011. Occurrence records were spatially aggregated on a 0.5° latitude×0.5° longitude grid and rigorously filtered according to criteria detailed in Jones *et al.* [40]. This minimised recording errors due to data being compiled from many sources and gave a binary value of presence or absence of each species for each cell.

### Projecting distribution shifts under climate change

A range of environmental oceanographic variables for predicting species' distributions were chosen, including bathymetry, sea surface temperature (SST), sea bottom temperature (SBT), salinity, sea ice concentration, primary productivity, and distance to coast. The DBEM used additional variables mentioned previously. Ocean oceanographic variables were interpolated onto a 0.5° latitude×0.5° longitude global grid using the nearest-neighbour method. Models were trained for each of 2 sets of average annual climatic data covering 1971–2000, the period corresponding as far as possible to the average climatic conditions over which occurrence data were compiled. For Maxent and Aquamap, predictions were subsequently projected into the future using a 30 year average centred on 2050. For DBEM, the model simulates changes in distribution over an annual time-step from 1971 to 2050. Environmental datasets (including physical variables as well as O_2_ concentration, pH and primary productivity) were obtained from Geophysical Fluid Dynamics Laboratory's Earth System Model (GFDL ESM2.1, [59]) and a further set of physical climate data (including SST, SBT, salinity and ocean advection) obtained from the World Climate Research Program (WCRP) Coupled Model Intercomparison Project phase 3 (CMIP3) multi-model dataset (http://esg.llnl.gov:8080). These data represented an ensemble of 12 different models that assessed by the fourth assessment of the Intergovernmental Panel on Climate Change (IPCC AR4), henceforth referred to as CMIP3-E. Both climatic datasets were modelled under the ‘high’ emission SRES A2 scenario and are thus characterised by a heterogenous world with a continuously increasing global population and regionally orientated economic development [60].

The changes in range of the seven threatened species were predicted under two scenarios of dispersal: no dispersal and full dispersal. Under the no dispersal scenario, distributions of the species were restricted to their predicted current range only and the species could not colonize areas outside its current distribution. In contrast, under the full dispersal scenario, a species' distribution could shift into all potentially suitable habitat using Maxent and AquaMaps and, in the case of DBEM, all suitable habitat within the projected dispersal range [33]. The scenario of no dispersal here represents a precautionary, conservative view and, following this assessment, the scenario of full dispersal is used throughout, agreeing with the observed ability of marine aquatic organisms to disperse under environmental change [61], [62].

A range of thresholds of minimum habitat suitability were applied to investigate the effect of excluding cells with lower levels of predicted habitat suitability on the analysis. Specifically, predicted habitat suitability values that are lower than the specific threshold equal 0. Thus, specific thresholds determine the extent of a species' most suitable (core) range. Thresholds are frequently used to transform the continuous predictions of relative suitability produced in species distribution modelling into predictions of presence/absence. There are several methods for selecting thresholds and their possible impacts on predicted distributions have been explored and discussed in the literature [63]–[65]. However, there is currently no consensus on the most suitable and stable method for applying thresholds to species' range projections [65]. As such, occurrence datasets for each species were split into 75% and 25% for model training and testing, respectively, and used to find the threshold that maximised accuracy of the model in predicting the observed occurrences/absences of a species (maximum training sensitivity plus specificity) [64]. This was implemented using the ROCR package in R [66]. Three fixed thresholds, of 0.05, 0.5 and 0.7, were applied to investigate the effect of increasingly restricting distributions and the implications for conclusions drawn from analysing the predicted distributions.

### Latitudinal centroids

Based on the results from the full dispersal scenario for each model, the average degree of range shift was calculated for each species in 2050 (average of 2036 to 2065) relative to 1985 (average of 1971 to 2000). This was done for each SDM, climatic dataset and each of the 4 thresholds and was calculated using an equation for distribution centroids, equation (1) [30]:
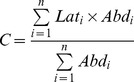
(1)where 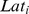 is the latitude of the centre of the spatial cell (*i*), *Abd* is the predicted relative abundance in the cell, and *n* is the total number of cells [30]. The difference between latitudinal centroids in projected and reference years was then calculated in kilometres (km) using equation (2) [38]:
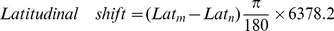
(2)


### Range overlap analysis

We used the degree of range overlap between the threatened species and the top 10 commercially targeted species in UK waters selected above as a proxy for investigating the degree of threat by overfishing through bycatch. We measured the potential overlap between the distributions of each threatened species with that of each targeted species using the Schoener's D index [67], [68], calculated by:
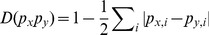
(3)where *p_x,i_ and p_y,i_* denote the probability assigned in a species distribution model computed for species *x* and *y* to grid cell *i* respectively.

This index quantifies the degree of overlap between two probability distributions or predictions of relative suitability, ranging from no overlap (0) to identical distributions (1), and is equivalent to the percent similarity index as proposed by Renkonen (1938) [67]. It has further been suggested as being best suited to computing niche overlaps from potential species' distributions [69]. A value of 0.1 was added to all 1985 values (D) to avoid extremely large percentage values caused by very low overlap in 1985 relative to the difference. The final overlap value thus represented the percentage difference in overlap relative to the 1985 value.

### Habitat suitability in protected areas

**Figure 1:**
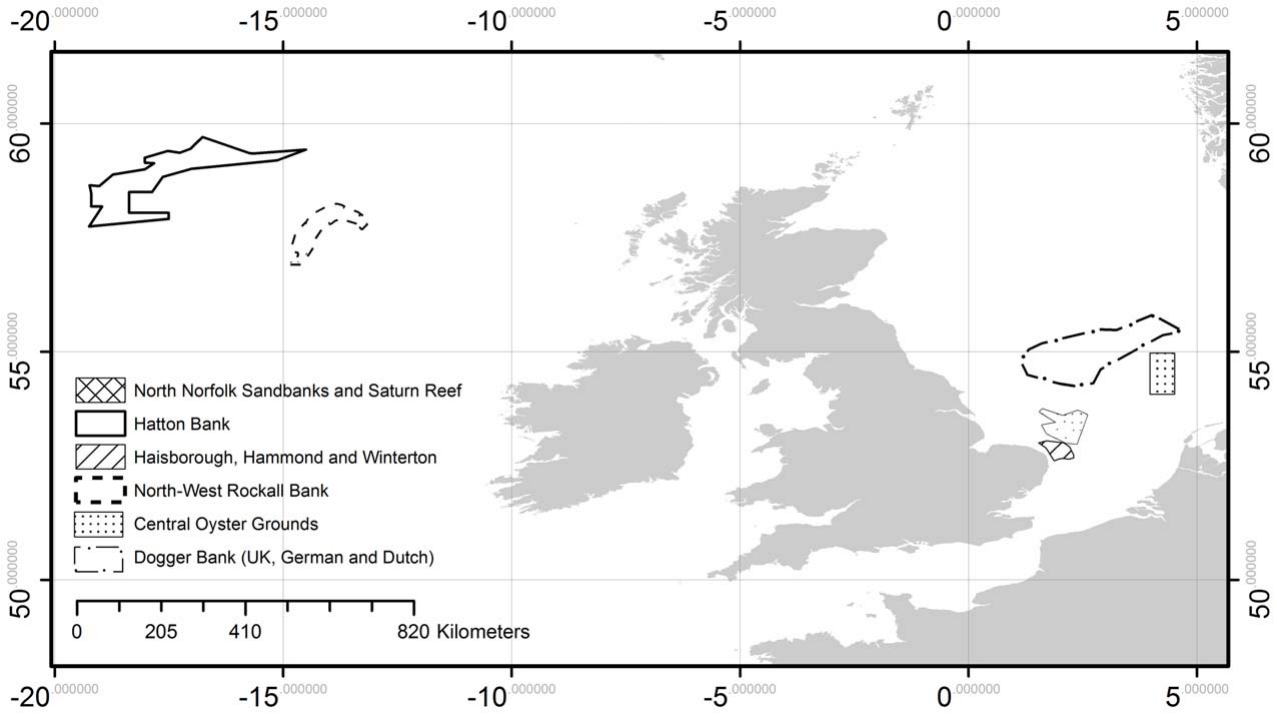
Candidate Special Areas for Conservation included in this study.

We calculated the changes in habitat suitability for the threatened species in candidate protected areas within and around the UK, Dutch or German waters. A set of candidate Special Areas of Conservation (cSACs) [70] that cover a range of habitat types and latitudinal distributions were selected. These sites were also chosen as being of appropriate size to the resolution of predicted species' distributions. Candidate SACs have been proposed but are yet to be adopted by the European Commission and formally designated by the local governments. They are designated for habitats and species listed on the Habitats Directive and include those areas considered to be in most need of conservation at a European level [71]. Under the Habitats Directive, Member States must take measures to maintain or restore natural habitats and wild species listed on the Annexes to the Directive at a favourable conservation status, introducing robust protection for habitats and species of European importance [71]. These cSACs include the Dogger Bank (UK, German and Dutch), Haisborough, Hammond and Winterton, together with North Norfolk Sandbanks and Saturn Reef (HHW & NNS), the Central Oyster Grounds (COG) (Dutch), North-West Rockall Bank, and Hatton Bank (Fig. 1.). Relative habitat suitability values of our sample of species for all grid cells within each cSAC were obtained for 1985 and 2050. The relative suitability values for each species were standardized for each model across all cSACs, resulting in a value scale between 0 and 1. The change in relative habitat suitability between 1985 and 2050 (2050 value – 1985 value) was calculated for each 0.5° latitude×0.5° longitude cell within a cSAC.

## Results

**Figure 2:**
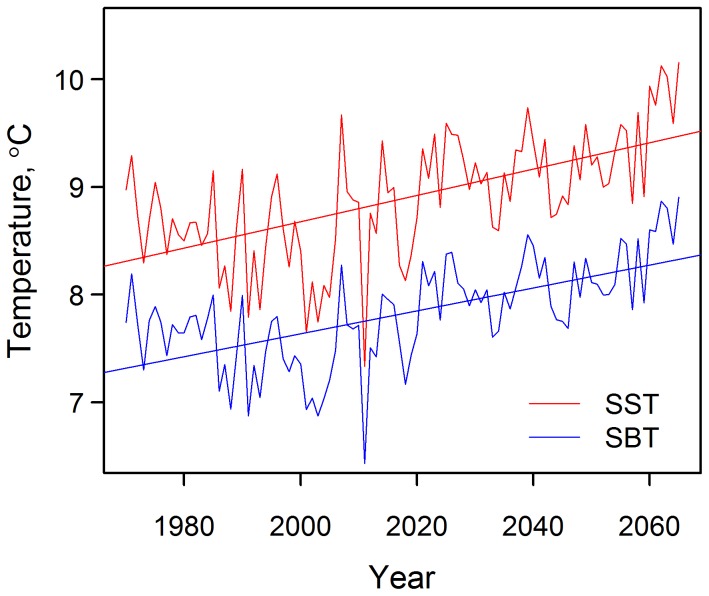
Temperature trends from 1970–2065 in the North Sea. Sea Surface Temperature (SST) and Sea Bottom Temperature (SBT) trends in the North Sea were averaged over all cells at a 0.5° latitude×0.5° longitude resolution.

Outputs from GFDL ESM2.1 suggest an average warming trend in the North Sea [72] from 1960 to 2065, with high interannual variability (Fig. 2). The pattern is similar for SST and SBT, which is to be expected given that the North Sea is relatively shallow (average depth ≈90 m). Average SST increases between 1985 and 2050 is 0.77°C and 1.27°C based on projections from GFDL ESM2.1 and CMIP3-E, respectively.

### Latitudinal centroids

**Figure 3:**
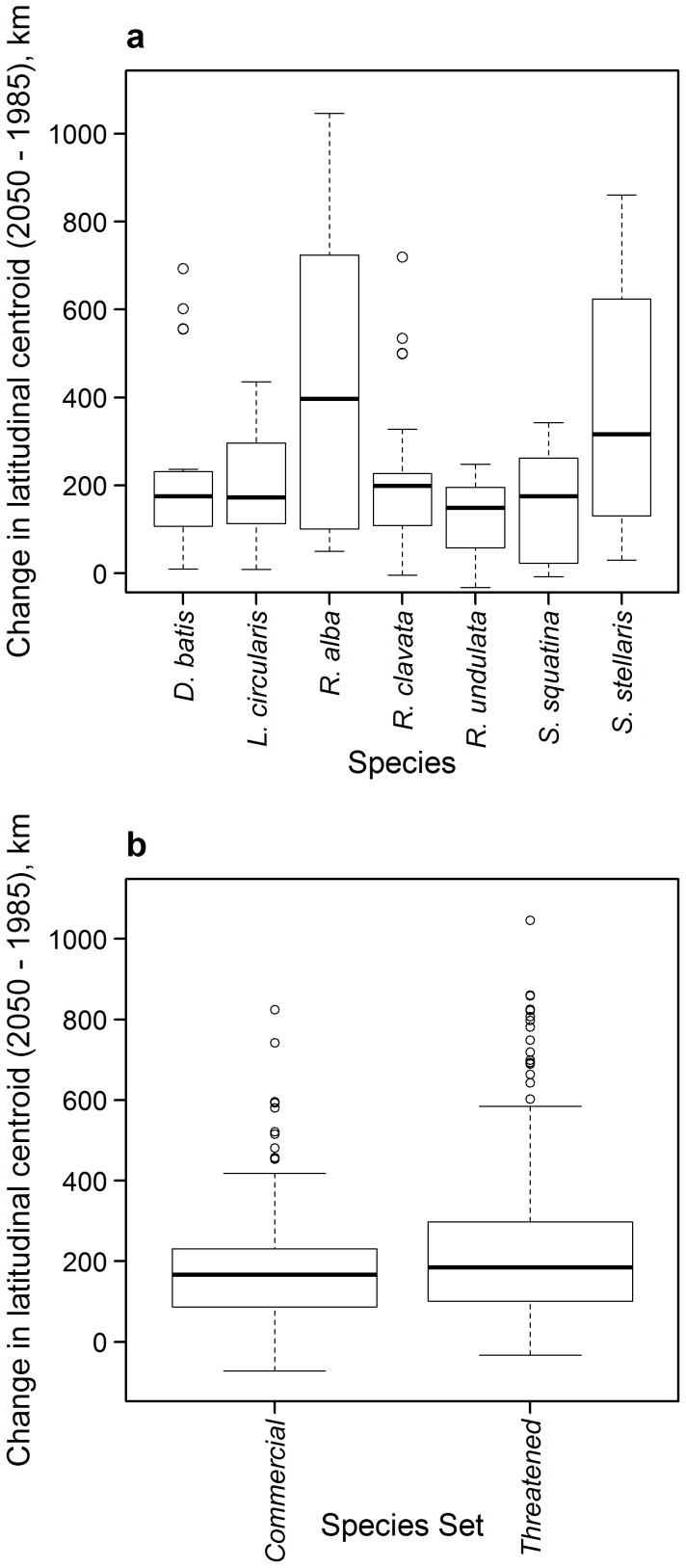
Shifts in latitudinal centroid for threatened and commercial species. Projected change (in km) in latitudinal centroid from 1985 to 2050 across the six SDM and climatic dataset combinations for a) each threatened species b) threatened and commercial species, grouped. Thick bars represent median values, the upper and lower ends of the box the upper and lower quartiles of the data, and the whiskers the most extreme datapoints no greater than 1.5 times inter-quartile range from the box. Points that are more extreme than whiskers are represented as circles.

Almost all models predicted northwards shifts in latitudinal centroid for the seven threatened species (Fig. 3a) and 10 commercially exploited species (Fig. 3b). Overall, our analysis projected that the distribution centroids of all species are expected to shift towards higher latitude from 1985 to 2050 under the SRES A2 scenario. The difference in poleward shift between commercially targeted and threatened species was not found to be significant when tested within each SDM model and climate dataset combination (two sample Wilcoxon test, p-value >0.05) (Fig. S1). The median projected rates of poleward range shift are 167.0 and 185.6 km over 65 years, corresponding to 26 and 28 km decade^−1^ for commercially exploited and threatened species, respectively (Fig. 3b). There is, however, variation within species predictions. For example, from 1985 to 2050, the predicted centroid distribution shift for *L. circularis* ranges from 8.9 km to 450 km northwards while that for *R. undulata* ranges from 32 km southwards to 247 km northwards. Contrasting these projections, three out of six SDM/GCM model combinations predict a >600 km northwards centroid shift for *S. stellaris* for the same period. *R. alba* was projected to shift at the fastest rate amongst the seven threatened species, reaching a maximum of 1046 km northwards by 2050 (threshold = 0.7). There is considerable variation in the predicted rate of range shift between SDMs, and to a lesser extent, between climate forcing used. However, no significant difference was found between latitudinal shifts projected using different SDM models within each of the two climate datasets, for both commercially targeted and threatened species (two sample Wilcoxon test, p-value>0.05).

### Predicted changes in range area

**Figure 4:**
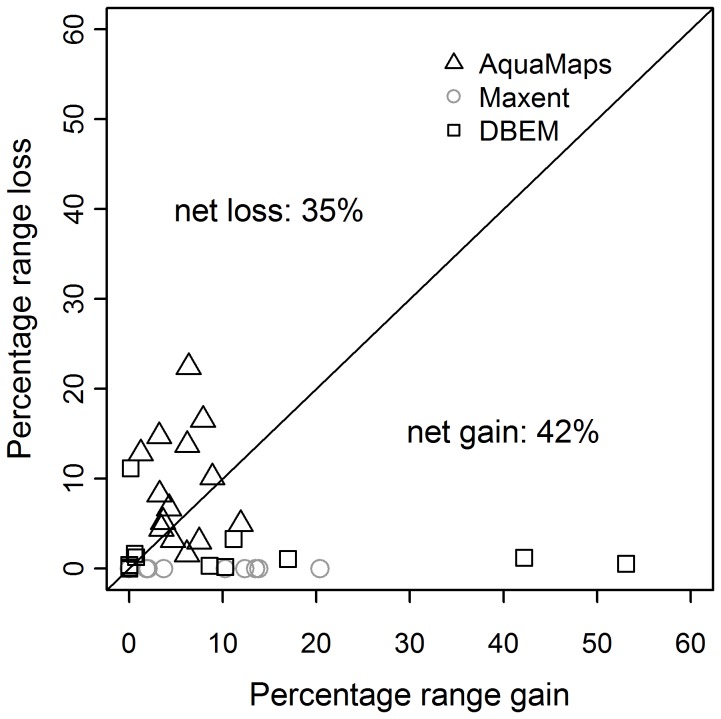
Changes in range area. Range loss and gain assuming no dispersal and full dispersal, respectively, between 1985 and 2050 for each SDM using GFDL and CMIP3-E climatic datasets.

Changes in area of predicted suitable habitat between 1985 and 2050 vary considerably, both between species and models (Fig. 4). Maxent and DBEM in general project net gains or no change in range area while AquaMaps frequently predicts net losses. More specifically, *L. circularis*, *R. clavata* and *S. stellaris*, were projected to have a net loss in range area by 2050 using 3 out of 6 model SDM-GCM combinations. While a net loss in range area is also seen in *R. alba* using the DBEM with CMIP3-E data, it contrasts the prediction with GFDL data that shows a net gain. The trend of predicted range area also varies between different climate forcing for *D. batis, S. squatina and R. alba*. Furthermore, the highest predicted gain (53.08%) and loss (22.44%) in area as a percentage of the 1985 range area were both predicted for *L. circularis*. The outlying points in Figure 4 are caused by *L. circularis* and *D. batis*, which are predicted to increase their range area by 53.08% and 42.17% respectively, using the DBEM model. These larger increases in range area are due to the DBEM- CMIP3-E model combination predicting greater range expansions to the northeast and West Atlantic than is seen for other models.

### Analysis of Range Overlap

**Figure 5:**
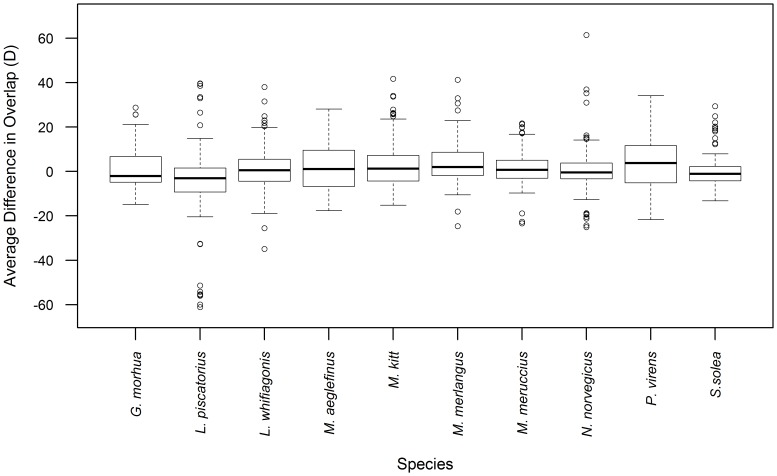
Changes in range overlap between species. Range of predicted changes in overlap (Schoener's D) as a percentage of 1985 overlap value for each commercial species with all threatened species. Values shown include all threatened species, SDMs, climatic datasets and thresholds.

The overall median change in range overlap between threatened and commercial species (expressed as a percentage of the 1985 overlap value), across models and thresholds, is relatively small (+/−4%) with the distribution of differences for threatened species showing no significant difference from 0 for *N. norvegicus*, *L. whiffiagonis*, *M. aeglefinus*, *M. kitt*, *M. merluccius* and *S. solea* (one-sample Wilcox test, p-value>0.05). However, selected model/threshold combinations projected large changes in overlap (exceeding +/−50%) (Fig. 5). All commercial species are predicted to decrease in overlap for at least one threatened species and modelling scenario. In contrast, all but two commercial species are, on average, projected to overlap more in predicted range with threatened species by 2050 (Table S1). The notable exception is *L. piscatorius*, which decreases in median overlap (median = −3.0%), particularly with *D. batis* (median = −0.7%, min = −61.1%) (Fig. S2a), *R. clavata* (median = −7.5%, min = −55.6) and *S. stellaris* (median = −5.8%, min = −51.7%). *R. alba* was projected to have the greatest increases in median range overlap across commercial species (mean = 4.9%). This species may thus be most likely to experience an increase in range overlap with the set of commercial species under climate change. *S. squatina*, on the other hand, was projected to have predominantly small, negative changes in median overlap across all commercial species (mean = −2.7%) and with only low variation between median values across species (−6%≤x≤1%) (Fig. S2b). *D. batis* shows a small average change in median values (0.1%) but also varies most across all commercial species (−61.1%<x<34.2%). The commercial species showing the maximum increase in range overlap by 2050 is *N. norvegicus* (61.4%, overlap with *R. alba*, using a 0.5 threshold).

### Change in relative suitability of key protected areas

**Figure 6:**
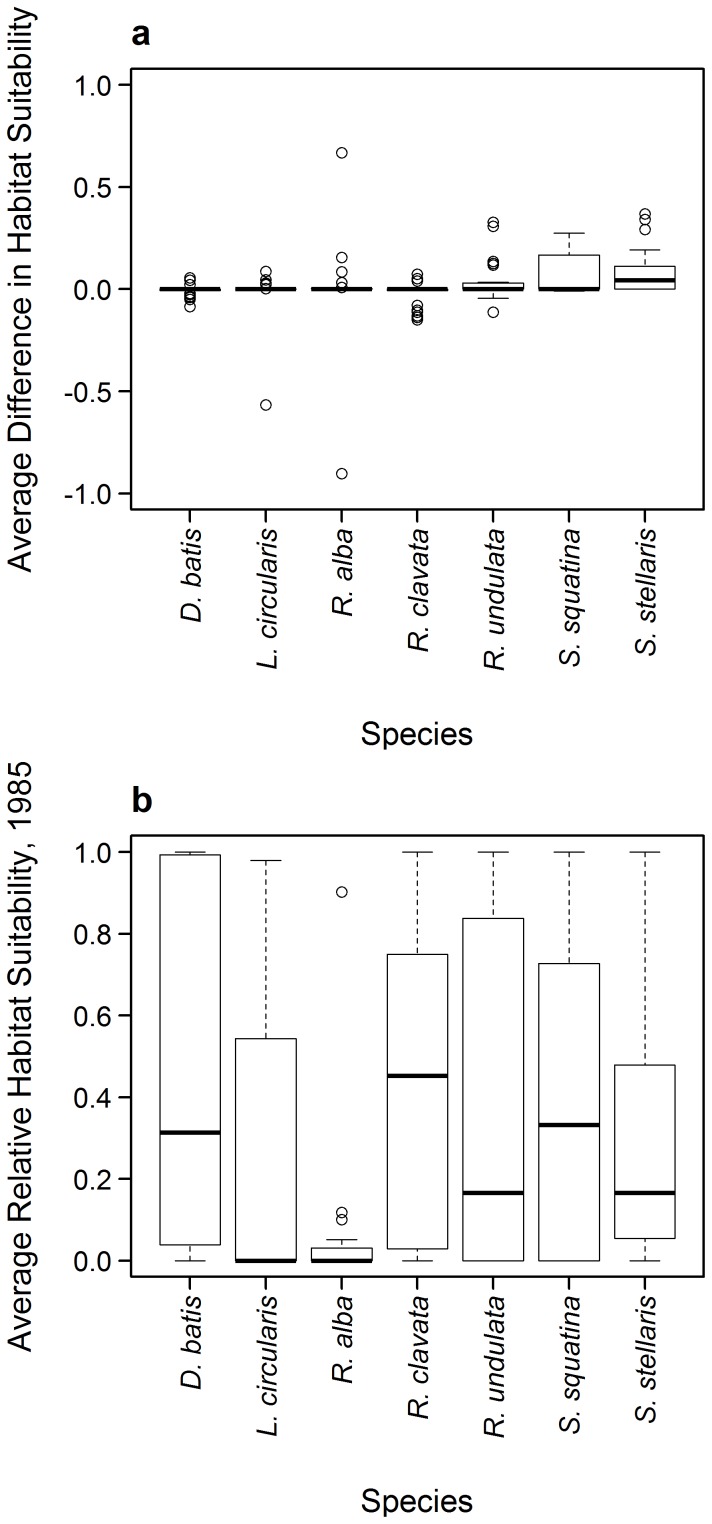
Habitat suitability in assessed candidate Special Area of Conservation (cSAC). a) Average difference in relative suitability (2050 – 1985), b) average relative habitat suitability values in 1985 for each threatened species in all assessed cSACs.

The overall average change in relative habitat suitability (RHS) over the protected areas is small, ranging between −0.03 and 0.09 from 1985 to 2050 (habitat suitability values lying between 0 and 1) (Fig. 6a). All species except *S. stellaris* were projected to have almost no median change in overall habitat suitability across all protected area sites. However, some species and SDM-GCM combinations show larger projected change in relative habitat suitability between 1985 and 2050. The greatest mean increase in RHS across all cSACs was, for example, projected for *S. stellaris* (0.08). This species, as well as *S. squatina*, with a mean increase in RHS of 0.06 and minimum prediction of −0.008, is thus likely to experience an average increase in habitat suitability over all the cSACs by 2050. These proposed increases are reflected in the Dogger Bank, with relatively consistently high and increasing relative habitat suitability values for *S. squatina and S. stellaris* across climate forcing and SDMs (Fig. S3). In contrast, *R. clavata* shows a median decrease in relative habitat suitability across all cSACs. Although averaging a small, positive change in relative habitat suitability (0.002), *R. alba* shows a wide range of variation.

Comparing the changes in absolute values of predicted habitat suitability in 1985 (Fig. 6b) is important as the impact of projected changes in RHS will likely depend on how suitable that habitat is currently for a particular species. For example, while the potential decrease in habitat suitability for *R. clavata* is accompanied by a mean habitat suitability in 1985 that is relatively high (0.43), the small potential increase (0.67) or decrease (−0.90) seen for *R. alba* accompanies a low average habitat suitability (0.05). A potential decrease in RHS may therefore have more adverse effect on *R. alba* than *R. clavata*. The broad range of RHS change observed for *R. alba* results from a strong predicted future increase in suitability of the Hatton Bank, using CMIP3-E data (Fig. S4), and a strong predicted decrease in suitability of the Rockall cSAC (Fig. S5). There are thus considerable variations in predictions between SDMs. This is highlighted in the case of *D. batis*, which shows consistent patterns of RHS across cSACs within the modelling procedures but variation in the values of RHS between models. *D. batis* is predicted to have highly suitable habitat and no future decrease in RHS in all SACs using AquaMaps (Table S2). Although positive, predictions for *D. batis* are generally lower in Maxent, showing an average decrease in the future. Using DBEM, suitability predictions of *D. batis* in 1985 are low or decreasing, other than in Rockall. Similar patterns of variation in trends predicted by the three SDMs were projected for *S. squatina* and *S. stellaris*. In general, habitat suitability for the threatened species in most SACs was projected to improve slightly under climate change. Specifically, habitats for threatened species in the Rockall cSAC are projected to improve in the future (Fig. S5).

### Sensitivity Analysis

**Figure 7:**
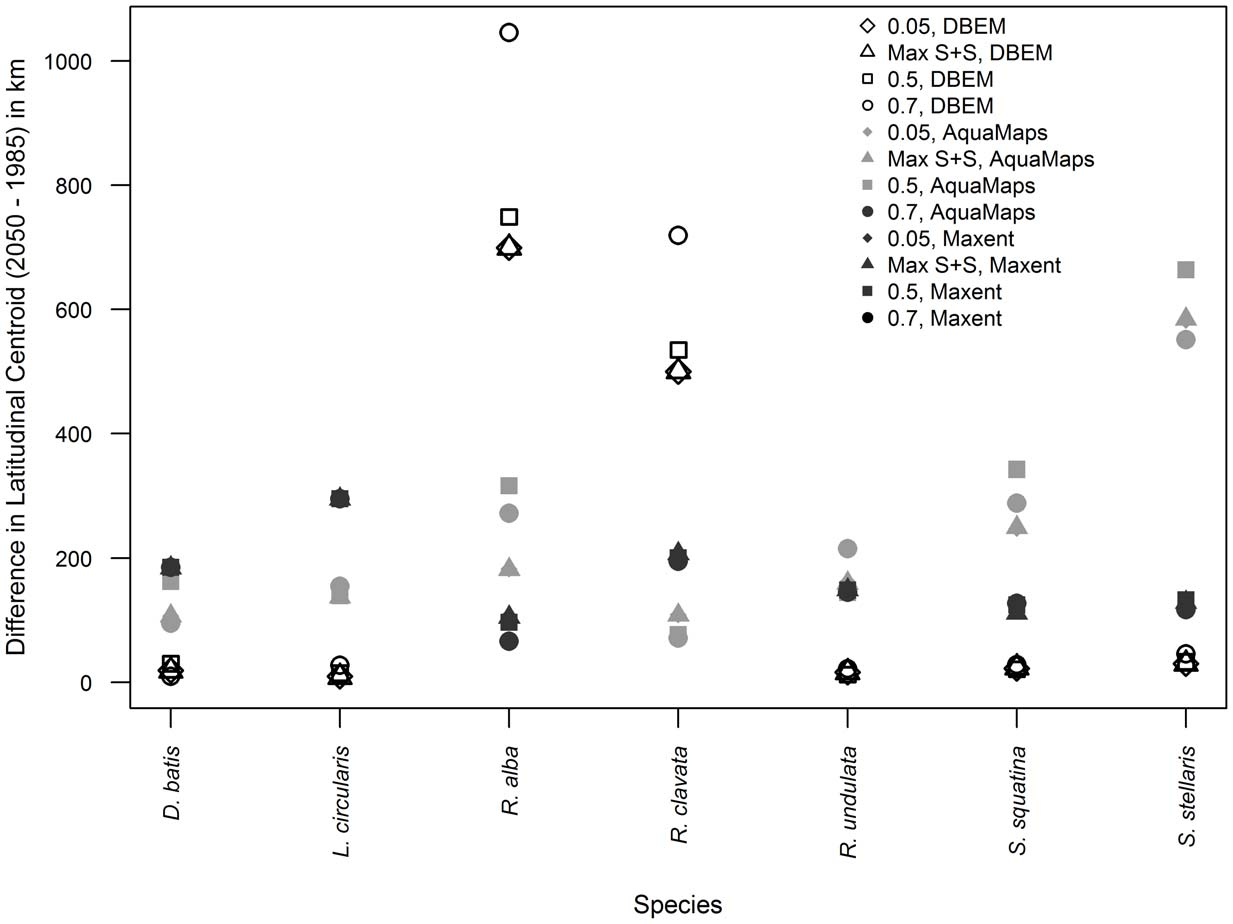
Latitudinal centroid change with thresholds. Difference in latitudinal centroids (2050 minus 1985 values, in km) using different threshold to restrict predictions made using AquaMaps, Maxent and DBEM. Thresholds applied include the three fixed thresholds (0.05, 0.5 and 0.7) and that that of maximum training sensitivity plus specificity (Max S+S).

The projected range shifts were generally robust to different threshold values, although variations in the projections between different thresholds are high for selected species (Fig. 7). A notable difference in latitudinal shift caused by applying different thresholds to 1985 and 2050 distribution is seen in *R. alba and R. clavata* using the DBEM model.

For the most part there is also strong agreement in the patterns of overlap values between threshold predictions, with more variation caused by differences in SDMs and GCMs. Variation in overlap change was frequently seen using a 0.7 threshold. For example, whereas the overlap of predicted ranges for *L. circularis* and *M. kitt* was predicted to increase by 11.4% of that in 1985, using a 0.7 threshold and averaged across SDMs and GCMs, this decreases to <2.5% when a larger range of habitat suitability area is taken into account. Conversely, overlap for *R. alba* and *S. solea* was predicted to increase by 4% to 6% using most thresholds but decrease by 1.3% when ranges were reduced using the most restrictive threshold (0.7).

## Discussion

Analyses and results presented here highlight the usefulness of using a multi-model approach in assessing climate change impacts on the distribution of marine species, given the variation in projections that can be obtained using different SDMs and GCMs in predicting species' distributions. For example, although differences between models in projecting northwards latitudinal shifts were not found to be significant, there are characteristic differences between predictions that reflect differences in model approaches and mechanisms. For example, the DBEM predicts a wider range of northwards movement across species, likely reflecting the incorporation of species specific values for intrinsic population growth, larval dispersal and adult migration. However, uncertainties and assumptions are inherent in any modelling procedure, in particular those projecting under novel, non-analogous climatic scenarios. It is therefore important to consider a range of plausible outcomes from multiple modelling appraoches. This corroborates studies modelling terrestrial species that proposes the use of a multi-model or ensemble approach for more robust predictions [73]. Here, general trends from a suite of model combinations as well as individual projections or outliers are considered and discussed.

### Latitudinal centroid shift

Our projected northward shifts in species' distributions supported the hypothesis for poleward shifts in response to climate change. They also agree with observed changes for marine species in the last few decades [53], [62], [74]. In particular, our projected rate of latitudinal centroid shifts corresponds well to observations in the North Sea [62], where, out of 36 species examined, six species showed boundary shifts in relation to both climate and time at a rate of 22 km decade^−1^. The projected rate of shift is smaller than that from a previous study that applies DBEM to model distribution shift of over 1000 species of marine fishes and invertebrates [30]. This difference is likely due to the inclusion of pelagic species by Cheung *et al.* [30], which are modelled using higher dispersal abilities in the DBEM model while the set of threatened species included in this study were all demersal, with lower dispersal abilities. As temperature gradients are dynamic and heterogeneous across the world, predicted rates of range shift will also vary according to the regions studied. The greater shift predicted here than observed for terrestrial species (0.6 km yr^−1^ [4]) was also expected due to the lower constraints on dispersal in the sea. Furthermore, two measures of thermal shifts used by Burrows *et al.* [75] showed that both the ‘velocity’ of climate change (the geographic shifts of isotherms over time) and the shift in seasonal timings of temperature to be higher in the ocean than on land at particular latitudes. The velocity of climate change was also less patchy in the sea than on land [75]. This disparity likely also accounts for greater observed and predicted distribution shifts seen in marine versus terrestrial species [4], [30], [62], [75].

### Changes in range area and overlap

Changes in range area under climate change may have important implications for species persistence. The association between patch area and extinction risk is one of the most ubiquitous observations in ecology [76] and has served as the basis for concepts central to conservation science, such as species area relationships, and population viability analysis. For example, one of the criteria employed by the IUCN Red List to define the level of threat (Criteria B) faced by a species is based on the extent of occurrence or area of occupancy [77]. Although it is frequently assumed that marine species have wide geographic ranges, 55% of skate species are endemic to single zoogeographic localities [78] and 70% have ranges spanning less than 20 degrees of latitude, a proxy for geographic range size [46]. Therefore, although results presented here did not show a marked climate-driven decrease in predicted range, contrary to projections for terrestrial species [15], [79], it would seem wise to take into account any potential decrease in range area and evaluate the range of predicted values rather than the median or mean.

While species are predicted to lose some of their range in at least one model prediction, the actual proportion of range being lost might also be informative, especially if more information on the dispersal capabilities and observed current distribution becomes available. While, for example, *S. squatina* is predicted by two models to reduce in overall range, given full dispersal, both values are relatively small. *D. batis*, on the other hand, is predicted to lose 11.6% of its current suitable habitat using one SDM/GCM model combination. However, the two Critically Endangered species assessed here, *L. circularis* and *D. batis*, may also experience net gains in suitable habitat, of 10.24% and 40.95% respectively with particular model combinations. The differing response of these two Critically Endangered species to climate change may thus likely depend on the relative dispersal ability of each species. For example, if *D. batis* is able to fully exploit potential new habitats it may overcome concurrent projected losses in suitable habitat. Overall, as both threatened and commercially exploited species were projected to shift northward simultaneously, the alteration in their overlap change was low except for selected species. Particularly, this study raises concern at increased threat from bycatch for *R. alba*, which potentially increases in overlap with all commercial species for at least one SDM/GCM combination.

### Protected area suitability

This study suggests that a change in climate will not result in an overall, unidirectional change in the relative habitat suitability of marine protected areas. This is generally because of the large variation in the predicted changes in relative habitat suitability between model combinations. Due to this variation across SDMs in assessing the likely protection afforded by a particular protected area to particular species, the magnitude of difference in relative habitat suitability across different SDMs and climate models seems of less importance than the actual identification of change in suitability by a model. Applying the precautionary principle, the possibility for decrease in habitat suitability of threatened species in protected areas should therefore be noted, thereby using the range of predictions to help identify the possible species and areas of concern.

Consistencies in patterns of the relative habitat suitability change between models for different SACs suggest that these inter-variations stem from characteristics of each modelling procedure, their mechanisms and algorithms. These differences might, for example, result in the majority of cells in a predicted distribution being given characteristically higher, or lower, values, explaining why predictions made using different climate forcing frequently show greater similarity than those made using the same climate forcing but different SDMs. Thus, a multi-model approach can capture structural uncertainty of projections in species distributions and suitability of candidate protected areas for particular species under climate change.

### Sensitivity and uncertainty

Analyses and results presented here highlight the variation in future projections that can be obtained using different SDMs and GCMs in predicting species' distributions. For a threatened species, variations in predictions may thus present the best and worst-case scenarios for the potential range under climate change. The variations in outputs are mainly driven by the algorithm by which the SDMs predicted species' distributions. For example, while the high habitat suitability values and equal weighting of variables in AquaMaps projections make this model less sensitive to temperature change, Maxent, which weights temperature as being the dominant predictor of distribution will be more sensitive to warming. As the relative response of species to change in one or other of the environmental variables and the possible interactions between them is highly uncertain, both projected responses should be considered. Thus, a multi-model or ensemble model approach helps quantify the variability in projections. In addition, the skill of a model in predicting changes in distribution could be assessed using model hindcasts and historical distribution data, rather than relying on the assumption that the models perform equally well in making future as current species distribution predictions. For example, comparison of historical projection of rate of range shift of exploited species in the Bering Sea and North Sea by DBEM showed a significant agreement between model outputs and observed rate of range shift [80]. Such model assessments could be applied to compare model preferences in future studies.

The implementation of a threshold value can often have a notable impact on conclusions drawn using species distribution or bioclimatic envelope models [50], [63], [65]. In this case, changes in latitudinal centroids were found to be robust to a range of thresholds. Alternative SDMs or climate forcing resulted in greater variations in our projections than the use of thresholds. Thus for this set of marine species, for which data paucity and reliability are an issue, the use of thresholds is not justified. The setting of thresholds would only allow reliable conclusions to be drawn if adequate data are available and a species is known to preferentially inhabit the most environmentally suitable habitat following range contraction from its historic distribution. Without sufficient data revealing the actual current of historical species distribution, all model outcomes were considered as equally valid, both in analysing latitudinal centroids and range overlaps.

A number of assumptions are made in Maxent, AquaMaps and the DBEM to deal with issues of data scarcity and quality that are especially common for marine organisms. Although data were rigorously controlled for quality to ensure maximum reliability (see [40]), the approaches do not incorporate ecological processes or biological interactions. Although the DBEM greatly advances the capabilities of modelling marine organisms in explicitly accounting for population growth and dispersal, none of the models account for predation pressure and food availability. As is common in bioclimatic envelope models, we also assume no adaptation to projected changes in environmental conditions.

A central criticism of species distribution and bioclimatic envelope modelling lies in the assumption that a species is in pseudo-equilibrium with its environment [35]. To ensure that this assumption was upheld here, all available valid occurrence data on each species was included to obtain as near as possible the species' absolute environmental tolerance limits. However, each of the species investigated here are thought to have been recently restricted to areas which do not adequately reflect their historic distribution for reasons other than change in environmental suitability, such as fishing and other human disturbances. Predictions made using these data are therefore unlikely to represent the actual current distribution of each species, potentially biasing estimates of a species' environmental tolerance limits and environmental envelopes. However, dated occurrence data recorded between 2000 and 2011 (ICES BTS surveys, including all beam trawl surveys) show that predicted distributions are within the historic distribution. Historic data thus supports the environmental tolerance limits and envelopes obtained using data obtained from a recently recorded distribution, following range contraction. Although range contraction may have consequences for the future dispersal of these species within patterns of suitable habitat, accurate hypotheses and conclusion could not be made due to lack of comprehensive sampling effort across the entire historic range in recent years. Future work could therefore involve a wider sampling across historic ranges and the compilation of a current observed dataset for each of these species.

Applying the precautionary principle, particularly for threatened species, it is advisable to consider the ranges of predictions in addition to the means, considering, for example, best and worst case scenarios. This is especially important for the two Critically Endangered species, *D. batis* and *S. squatina*, for which the ability to respond to climatic change or novel threats is expected to be limited by their putative restriction as small populations in areas which are not optimal and from which dispersal might be limited. Species that have shifted in distribution or increased in abundance in warmer years have previously been observed to be those with faster life history traits, with smaller body sizes, faster maturation and smaller sizes at maturity [62], [74]. This result would be expected if the difference in rate of movement shown by particular taxa resulted from differential rates of population turnover. The threatened species assessed here are, however, characterised by slower life history traits, with larger sizes and later maturation rates than most species in the commercially exploited group, yet their environmental envelope is shown to shift more. If dispersal and distribution shift are linked to life history traits, even though threatened species are here predicted to show a greater median northward shift than commercial species, whether they actually will be able to disperse to occupy predicted potential ranges is unknown. The study of these species and the threat to them posed by climate change would therefore benefit from an assessment of their observed shift over time and their capacity to disperse and whether or not this might be promoted by the implementation of particular protected areas. Further work should also assess the variation in outputs produced by a range of emission scenarios under SRES or RCP (Representative Concentration Pathways, developed for the IPCC 5^th^ Assessment Report).

## Conclusion

Evaluating the possible effects of climatic change on species' distributions using bioclimatic envelope models is a useful tool to gain insight on how species might respond under future climatic change. In particular, the ability to make this assessment for threatened species marks an important contribution amid calls for conservation planning to take an adaptive response to enhance the resilience of protected areas and the biodiversity within them to climate change. Although all species investigated in this study are predicted to move northwards by 2050, the effect of climate change on range areas and the suitability of a set of protected areas for this set of threatened species is less detrimental than would be expected based upon studies of similar changes in the terrestrial environment. This study highlights the variation in future projections according to the SDM and GCM used. As variation stems from characteristics of the models themselves, projections from multiple models better capture model uncertainties and allow identification of a best and worst case scenario of change. For critically endangered species and those facing high levels of threat, it is particularly important to apply the precautionary principle. In the marine environment, there exist many unknowns and uncertainties concerning species, their habitats and the threats they face. A multi-model approach enables a precautionary approach when considering the persistence of threatened species given their uncertain responses to future climate change.

## Supporting Information

Figure S1 **Shifts in latitudinal centroid for threatened and commercial species.** Projected change (in km) in latitudinal centroid from 1985 to 2050 using each of the six SDM and climatic dataset combinations, for both threatened species and commercial species. Thick bars represent median values, the upper and lower ends of the box the upper and lower quartiles of the data, and the whiskers the most extreme datapoints no greater than 1.5 times inter-quartile range from the box. Points that are more extreme than whiskers are represented as circles.
(TIF)

Figure S2 **Difference in overlap between species.** Difference in range overlap, (Schoener's D) as a percentage of the 1985 overlap value, between commercial species and a) *Dipturus batis* b) *Squatina squatina*. Thick bars represent median values, the upper and lower ends of the box the upper and lower quartiles of the data, and the whiskers the most extreme datapoints no greater than 1.5× inter-quartile range from the box. Points that are more extreme than whiskers are represented as circles.
(TIF)

Figure S3 **Differences in habitat suitability for threatened species in the Dogger Bank.** Difference in habitat suitability for the each of the six SDM/GCM combinations. Difference (2050 – 1985 values) in relative habitat suitability was calculated following standardization across all cSACs for each species and model.
(TIF)

Figure S4 **Differences in habitat suitability for threatened species in Hatton Bank.** Difference in habitat suitability for the each of the six SDM/GCM combinations. Difference (2050 – 1985 values) in relative habitat suitability was calculated following standardization across all cSACs for each species and model.
(TIF)

Figure S5 **Differences in habitat suitability for threatened species in Rockall.** Difference in habitat suitability for the each of the six SDM/GCM combinations. Difference (2050 – 1985 values) in relative habitat suitability was calculated following standardization across all cSACs for each species and model.
(TIF)

File S1 **Supplementary Methods.**
(DOCX)

Table S1 **Median difference in range overlap, (Schoener's D) as a percentage of the 1985 overlap value, between threatened and commercial species.** Minimum, maximum and average overlap values are given for threatened species and average and overall median overlap values for commercial species.
(PDF)

Table S2 **Habitat Suitability values in 2000 and differences (2050 – 2000) for** ***D. batis*** **in all cSACs for each SDM/GCM combination.**
(PDF)
