# Supplementary figures and images for: Predicting the Impact of Climate Change on Threatened Species in UK Waters
Source: PLoS One. 2013 Jan 22;8(1):e54216. doi: 10.1371/journal.pone.0054216 (PMC3551960; doi:10.1371/journal.pone.0054216)

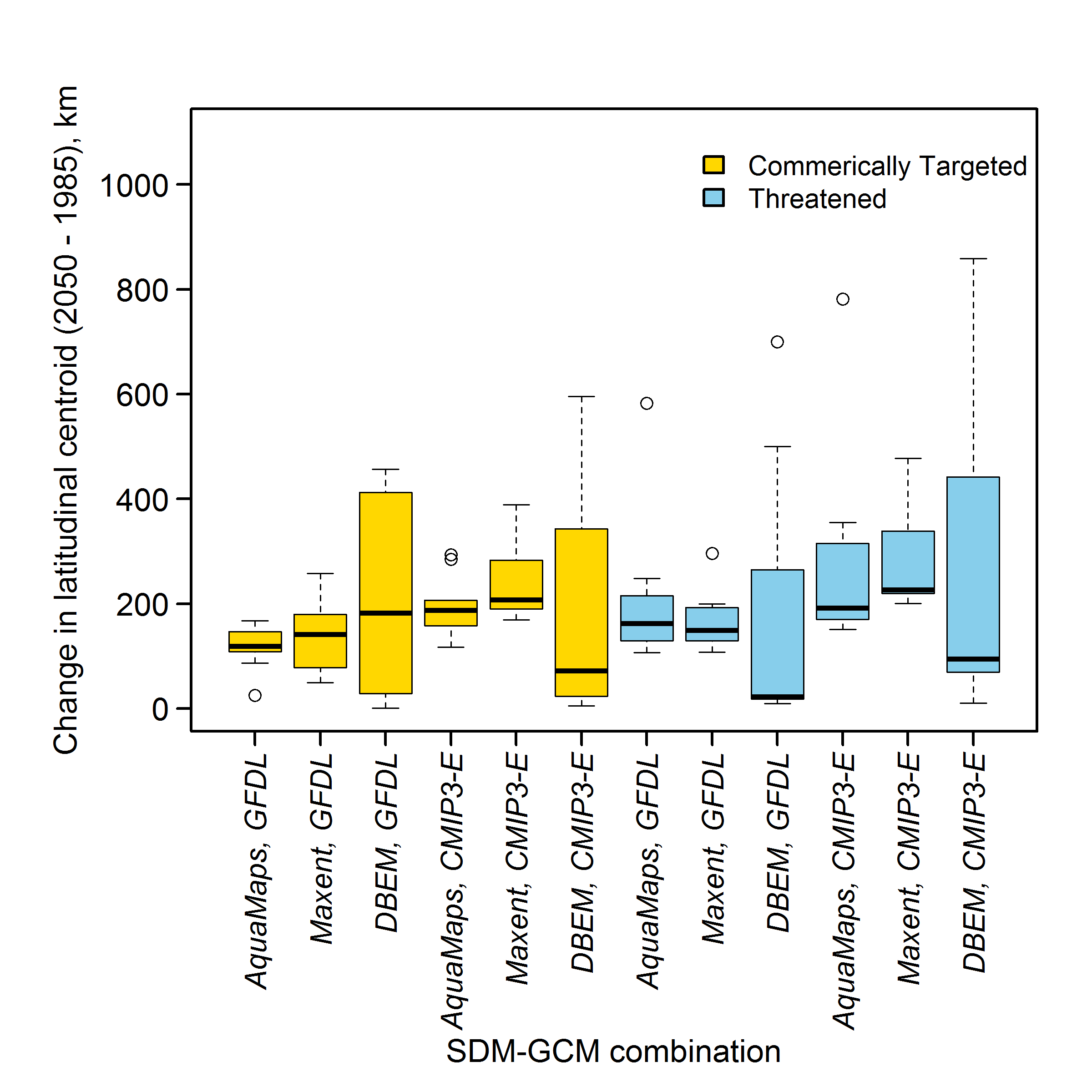

Supplement: Figure S1 — Shifts in latitudinal centroid for threatened and commercial species. Projected change (in km) in latitudinal centroid from 1985 to 2050 using each of the six SDM and climatic dataset combinations, for both threatened species and commercial species. Thick bars represent median values, the upper and lower ends of the box the upper and lower quartiles of the data, and the whiskers the most extreme datapoints no greater than 1.5 times inter-quartile range from the box. Points that are more extreme than whiskers are represented as circles. (TIF) [file pone.0054216.s001.tif]

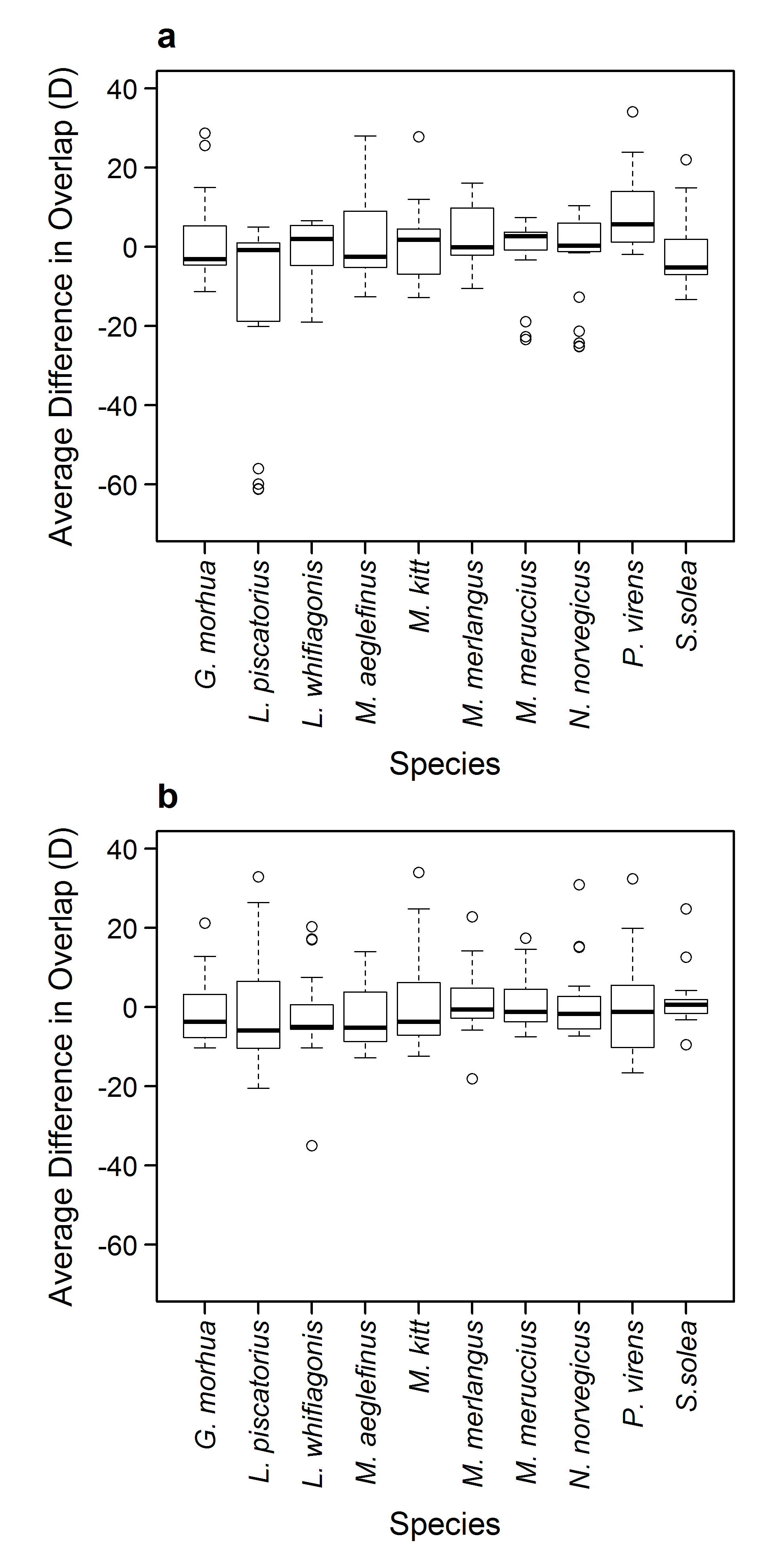

Supplement: Figure S2 — Difference in overlap between species. Difference in range overlap, (Schoener's D) as a percentage of the 1985 overlap value, between commercial species and a) Dipturus batis b) Squatina squatina. Thick bars represent median values, the upper and lower ends of the box the upper and lower quartiles of the data, and the whiskers the most extreme datapoints no greater than 1.5× inter-quartile range from the box. Points that are more extreme than whiskers are represented as circles. (TIF) [file pone.0054216.s002.tif]

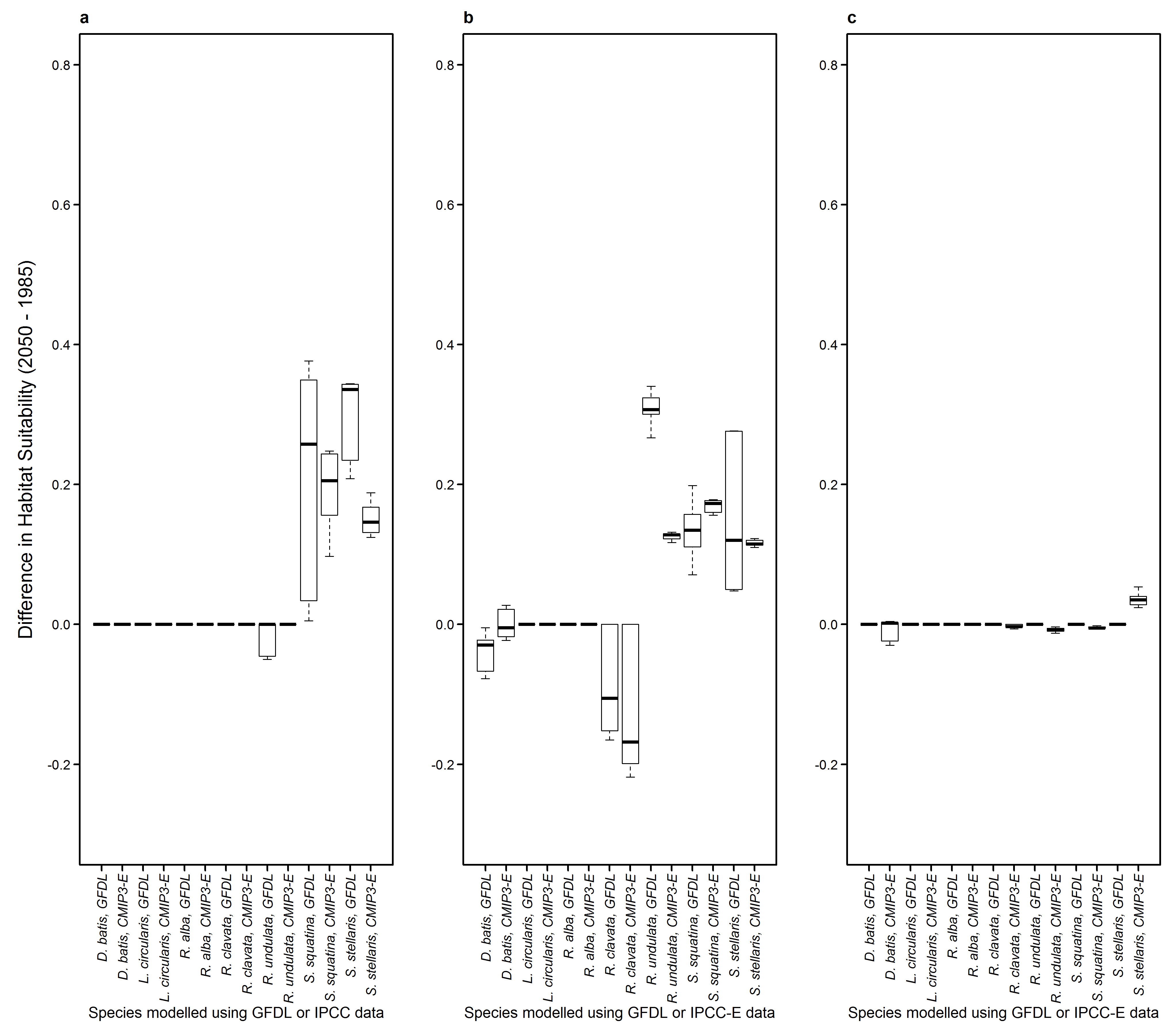

Supplement: Figure S3 — Differences in habitat suitability for threatened species in the Dogger Bank. Difference in habitat suitability for the each of the six SDM/GCM combinations. Difference (2050 – 1985 values) in relative habitat suitability was calculated following standardization across all cSACs for each species and model. (TIF) [file pone.0054216.s003.tif]

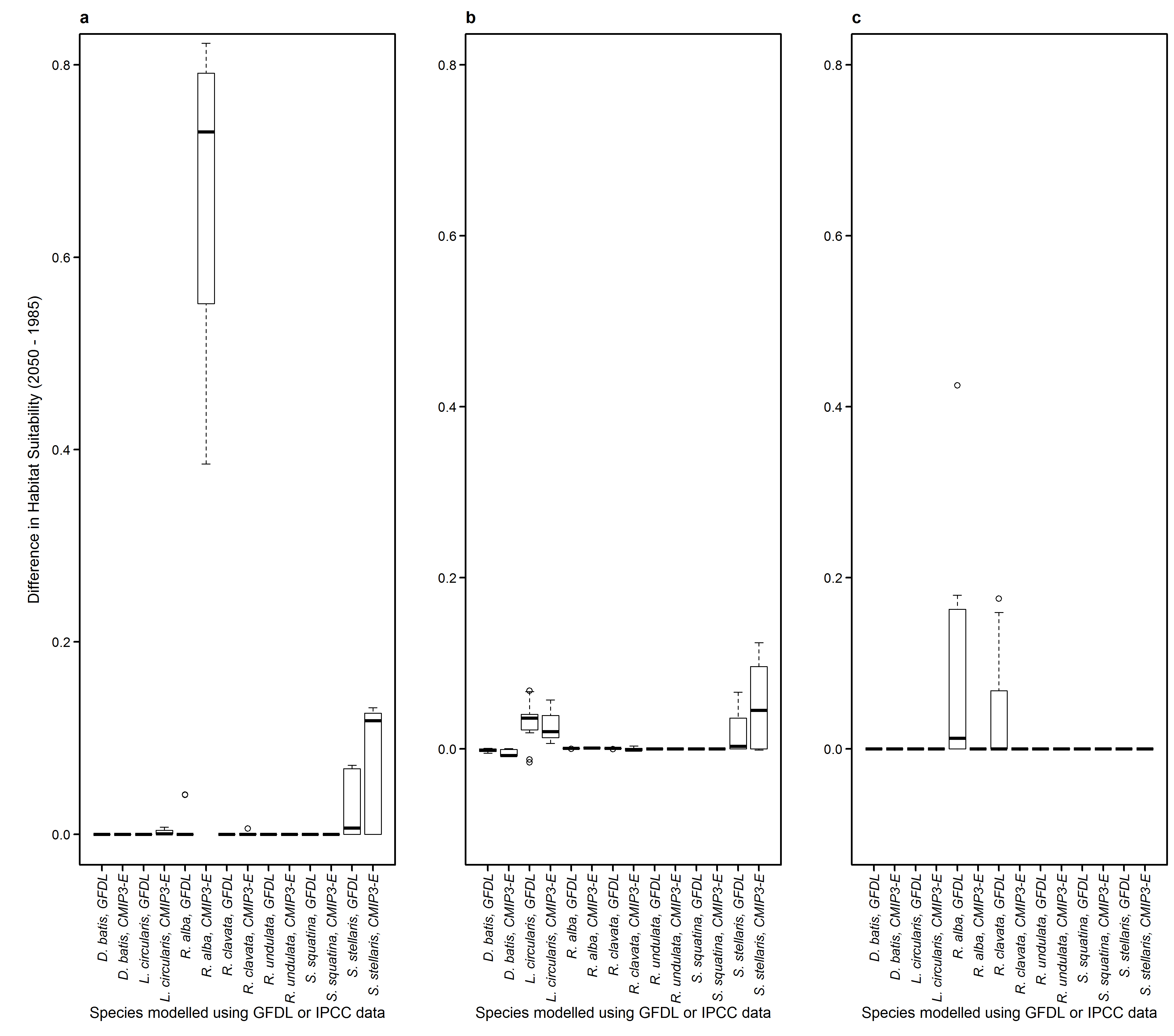

Supplement: Figure S4 — Differences in habitat suitability for threatened species in Hatton Bank. Difference in habitat suitability for the each of the six SDM/GCM combinations. Difference (2050 – 1985 values) in relative habitat suitability was calculated following standardization across all cSACs for each species and model. (TIF) [file pone.0054216.s004.tif]

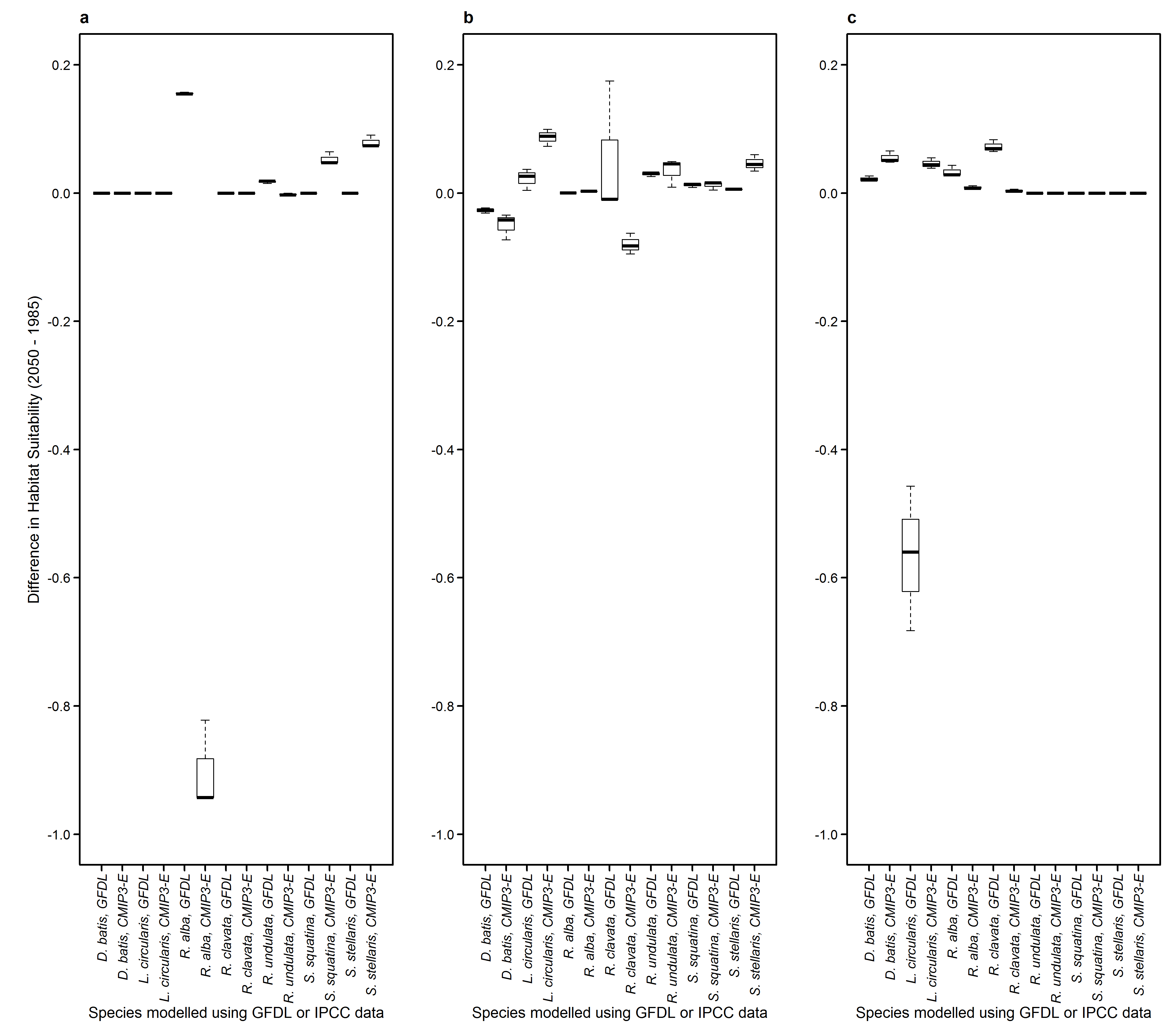

Supplement: Figure S5 — Differences in habitat suitability for threatened species in Rockall. Difference in habitat suitability for the each of the six SDM/GCM combinations. Difference (2050 – 1985 values) in relative habitat suitability was calculated following standardization across all cSACs for each species and model. (TIF) [file pone.0054216.s005.tif]
