# Supplementary material for: Predicting the Impact of Climate Change on Threatened Species in UK Waters
Source: PLoS One. 2013 Jan 22;8(1):e54216. doi: 10.1371/journal.pone.0054216 (PMC3551960; doi:10.1371/journal.pone.0054216)
Supplement: File S1 — Supplementary Methods. (DOCX) [file pone.0054216.s006.docx]

**Supplementary Methods**

**Maxent**

Maxent [1] uses a complex generative approach [2] to estimate the environmental co-variates conditioning species’ presence from presence only occurrence data and a suite of environmental variables. The final prediction is based on the principle of maximum entropy, which specifies that the best approximation of an unknown distribution is the probability distribution with maximum entropy, subject to the constraints imposed by the sample of species’ presence observations [2]. Maxent has been shown to compete well with alternative approaches in terms of model testing statistics [2,3] and is robust to small sample sizes [4]. Models were constructed using Maxent version 3.3.3e with default parameters for a random seed, regularization parameter (1, included to reduce over-fitting), maximum iterations (500), convergence threshold (0.00001) and maximum number of background points (10000 points which have not been recorded as present). The relative contribution of environmental variables to each iteration of the model was also carried out automatically. The model trained on the set of environmental variables representing the current time period was then ‘projected’ by its application to a set of the same environmental variables representing future climate.

**AquaMaps**

In contrast, the AquaMaps approach to modelling species’ distributions uses simple, numerical descriptors of species’ relationships with environmental variables to predict distributions from occurrence databases. Environmental envelopes for each variable are calculated by associating occurrence data with current environmental variables to find the absolute and ‘preferred’ preference ranges (calculated as shown in [5,6]), with the relationship between species’ occurrence and environmental limits is specified by a trapezoidal distribution [5–7]. While relative environmental suitability is therefore assumed to be uniformly high through the preferred parameter range (with a probabliy of 1), values lying outside the observed minimum and maximum are assigned a probability of 0. Suitability decreases linearly between the two thresholds. Predicted current distributions/ habitat suitability are generated multiplicatively from a suite of ‘environmental envelopes’ over each cell in a study area. Resulting cell values lie between 0 and 1 and represent the relative suitability of that cell for the specified species. The environmental envelopes obtained for a 30 year centred average on 1985 were then applied to environmental datasets representing future climatic scenarios. This methodology does not allow complex, non-linear interactions to be fit between predictors and assumes they carry equal weight in predictions. Although simple, AquaMaps allows better transparency and understanding in the wider, non-modelling, community while also explicitly promoting incorporation of expert judgement.

Expert opinion was incorporated into Maxent and AquaMaps to refine predictions by eliminating (‘clipping’) areas. Area eliminated were those outside known occurrence ranges, reported occurrence/absence in large ocean basins [delineated by the United Nations’ Food and Agricultural Organisation (FAO) statistical area, [www.fao.org/fishery/area/search/en](http://www.fao.org/fishery/area/search/en)] or beyond species specific depth limits [6]. This avoided over-prediction of relative habitat suitability in areas of the world where species are known not to occur, or which are unsuitable due to depth, the limits of which may be over-estimated in Maxent and AquaMaps due to the relatively low resolution of depth and occurrence data, in particular at the edge of the continental shelf. Maximum depth limits obtained from Fishbase [8] were increased by 50% in predictions for both time periods. This allowed for the deepening of species with ocean warming that has been observed [9] while preventing difference in predictions between the two time periods being inflated and biased by applying different depth cut off points.

**Dynamic Bioclimate Envelope Model (DBEM)**

Contrasting the above approaches, the Dynamic Bioclimate Envelope Model (DBEM) [10–12] combines statistical and mechanistic approaches in predicting species’ distributions. Firstly, we employed the *Sea Around Us Project* (<http://www.seaaroundus.org/>) (SAUP) model, which was developed to address the need for distributional ranges of commercial fish and invertebrates for mapping global fisheries catches and studying the impacts of fisheries on the world’s marine ecosystems. The SAUP model [13] applies a set of environmental ‘filters’ to delimit a species’ current distribution to a realistic range from a potentially global distribution. Filters use known geographic or environmental tolerance limits to restrict a species’ potential distribution and were obtained for FAO areas, latitudinal limits (with a further range-limiting filter preventing occurrence in semi-enclosed seas) and depth limits and habitat preference. For further details on the application of filters see [6,13]. From this distribution, the DBEM defines the species’ bioclimatic envelope by its ‘preference profile’ (the relative suitability of different environmental values) for each environmental variable. Preference profiles were thus created by overlaying environmental data from 1971 – 2000 with maps of current relative abundance produced using the *Sea Around Us Project* model [6,13]. Variables incorporated into the DBEM include sea surface temperature, sea bottom temperature, coastal upwelling, salinity, distance from sea-ice and habitat types (coral reef, estuaries and sea mounts) (for full description see [11,12]).

The DBEM differs from other Bioclimatic Envelope Models (BEMs) in simulating changes in a species’ relative abundance by incorporating a logistic population growth model [10] as well as ecophysiological parameters. First of all, the model simulates how changes in temperature, oxygen content (represented by O_2_ concentration) and pH would affect fish and invertebrate growth, determined by the difference between anabolism and catabolism [12], using an algorithm derived from the von Bertalanffy growth function (VBGF) [14]. The VBGF parameters are subsequently used to determine change in carrying capacity in each 0.5° latitude × 0.5° longitude cell. Carrying capacity is expressed as a function of recruitment and expected biomass per recruit, the later being determined using a size-based population model. The model assumes a population’s spatial and temporal dynamics to be determined by its species specific intrinsic population growth, larval dispersal and adult migration [10,12]. Larval dispersal and adult migration are assumed to follow ocean currents, with the distance and direction of movement a function of predicted pelagic larval duration (based on an empirical equation [15]). Intrinsic population growth rate is dependent on the growth rate and carrying capacity of the species, which is determined by the species’ environmental preference profile and the resulted habitat suitability. Habitat suitability is expected to be positively correlated with carrying capacity for each species. Carrying capacity values for each environmental predictor are then combined multiplicatively to obtain a final value of habitat carrying capacity for a species.

**References**

1. Phillips SJ, Dudík M, Schapire RE (2004) A maximum entropy approach to species distribution modeling. Proceedings of the twenty-first International Conference on Machine Learning, ACM Press, New York: 655 - 662.

2. Phillips SJ, Anderson RP, Schapire RE (2006) Maximum entropy modeling of species geographic distributions. Ecological Modelling 190: 231–259.

3. Elith J, Ferrier S, Guisan A, Graham CH, Anderson RP, et al. (2006) Novel methods improve prediction of species’ distributions from occurrence data. Ecography 29: 129–151.

4. Pearson RG, Raxworthy CJ, Nakamura M, Townsend Peterson A (2007) Predicting species distributions from small numbers of occurrence records: a test case using cryptic geckos in Madagascar. Journal of Biogeography 34: 102–117.

5. Kaschner K, Watson R, Trites A, Pauly D (2006) Mapping world-wide distributions of marine mammal species using a relative environmental suitability (RES) model. Marine Ecology Progress Series 316: 285–310..

6. Jones MC, Dye SR, Pinnegar JK, Warren R, Cheung WWL (2012) Modelling commercial fish distributions: Prediction and assessment using different approaches. Ecological Modelling 225: 133–145.

7. Ready J, Kaschner K, South AB, Eastwood PD, Rees T, et al. (2010) Predicting the distributions of marine organisms at the global scale. Ecological Modelling 221: 467–478..

8. Froese R, Pauly D (2011) Fishbase. World Wide Web electronic publication. Available:www.fishbase.org, version (02/2011). Accessed 2 February 2012.

9. Dulvy NK, Rogers SI, Jennings S, Stelzenmller V, Dye SR, et al. (2008) Climate change and deepening of the North Sea fish assemblage: a biotic indicator of warming seas. Journal of Applied Ecology 45: 1029–1039.

10. Cheung WWL, Lam VWY, Pauly D (2008) Modelling present and climate-shifted distributions of marine fishes and invertebrates. Fisheries Centre Research Report 16.

11. Cheung WWL, Lam VWY, Sarmiento JL, Kearney K, Watson R, et al. (2009) Projecting global marine biodiversity impacts under climate change scenarios. Fish and Fisheries 10: 235–251..

12. Cheung WWL, Dunne J, Sarmiento JL, Pauly D (2011) Integrating ecophysiology and plankton dynamics into projected maximum fisheries catch potential under climate change in the Northeast Atlantic. ICES Journal of Marine Science 68: 1008–1018.

13. Close C, Cheung W, Hodgson S, Lam V, Watson R, et al. (2006) Distribution ranges of commercial fishes and invertebrates. Fisheries Centre Research Report 14: 27 - 37.

14. von Bertalanffy L (1951) Theoretische Biologie. Zweiter Band, Stoffwechsel, Wachstum. Bern: Z. Francke. p.

15. O’Connor MI, Bruno JF, Gaines SD, Halpern BS, Lester SE, et al. (2007) Temperature control of larval dispersal and the implications for marine ecology, evolution, and conservation. Proceedings of the National Academy of Sciences of the United States of America 104: 1266–1271.
