# Supplementary material for: Predicting the Impact of Climate Change on Threatened Species in UK Waters
Source: PLoS One. 2013 Jan 22;8(1):e54216. doi: 10.1371/journal.pone.0054216 (PMC3551960; doi:10.1371/journal.pone.0054216)
Supplement: Table S1 — Median difference in range overlap, (Schoener's D) as a percentage of the 1985 overlap value, between threatened and commercial species. Minimum, maximum and average overlap values are given for threatened species and average and overall median overlap values for commercial species. (PDF) [file pone.0054216.s007.pdf]

	G. morhua	*L. piscatorius*	*L. whiffiagonis*	*M. aeglefinus*	*M. kitt*	*M. merlangus*	*M. merluccius*	*N. norvegicus*	*P. virens*	*S. solea*	Min value	Max value	Average across species
D. batis	-3.0	-0.7	2.0	-2.5	1.8	-0.1	2.8	0.4	5.8	-5.2	-61.1	34.2	0.1
L. circularis	-2.8	-1.5	-0.6	3.9	0.8	7.5	0.7	1.1	7.5	0.3	-32.6	41.7	1.7
R. alba	8.3	-2.6	5.5	9.7	6.0	9.5	3.3	0.3	9.8	-0.8	-25.5	61.4	4.9
R. clavata	-2.2	-7.5	0.5	-1.6	1.5	1.4	-2.7	-0.9	-1.7	-1.3	-55.6	33.0	-1.4
R. undulata	-4.7	0.2	-0.7	-5.5	-4.5	-0.6	3.2	-0.9	-3.1	-2.9	-32.7	31.5	-1.9
S. Squatina	-3.7	-5.8	-5.0	-5.2	-3.6	-0.6	-1.1	-1.6	-1.2	0.7	-34.9	34.0	-2.7
S. stellaris	6.4	-5.8	-4.4	3.7	1.2	-0.1	-0.6	-1.0	7.1	1.1	-51.7	33.21	0.8
Total average	1.3	-5.5	1.1	1.8	2.7	3.8	1.3	0.8	3.7	-0.2	-34.9	34.0	1.1
Total Median	-2.03	-3.03	0.59	1.14	1.25	1.97	0.75	-0.43	3.79	-1.04			
