# Supplementary material for: Predicting the Impact of Climate Change on Threatened Species in UK Waters
Source: PLoS One. 2013 Jan 22;8(1):e54216. doi: 10.1371/journal.pone.0054216 (PMC3551960; doi:10.1371/journal.pone.0054216)
Supplement: Table S2 — Habitat Suitability values in 2000 and differences (2050 – 2000) for D. batis in all cSACs for each SDM/GCM combination. (PDF) [file pone.0054216.s008.pdf]

SDM	AquaMaps	AquaMaps	Maxent	Maxent	DBEM
GCM	GFDL	CMIP3-E	GFDL	CMIP3-E	GFDL
	1985 values				
Dogger Bank	1.000	1.000	0.414	0.230	0.000
	1.000	1.000	0.404	0.230	0.000
	1.000	1.000	0.444	0.283	0.000
	1.000	1.000	0.464	0.282	0.000
	1.000	1.000	0.416	0.230	0.000
	1.000	1.000	0.412	0.230	0.000
	1.000	1.000	0.549	0.295	0.000
	1.000	1.000	0.497	0.288	0.000
	1.000	1.000	0.493	0.289	0.000
	1.000	1.000	0.449	0.287	0.000
	1.000	1.000	0.386	0.262	0.000
	1.000	1.000	0.521	0.291	0.000
Central Oyster Grounds	1.000	1.000	0.354	0.251	0.000
	1.000	1.000	0.368	0.251	0.000
Haisborough, Hammond and Winterton and	1.000	1.000	0.492	0.231	0.000
North Norfolk Sandbanks and Saturn Reef	1.000	1.000	0.521	0.307	0.000
	1.000	1.000	0.501	0.234	0.000
	1.000	1.000	0.492	0.228	0.000
Hatton Bank	0.854	0.854	0.061	0.043	0.000
	0.871	0.871	0.062	0.043	0.000
	0.881	0.881	0.071	0.051	0.000
	0.906	0.906	0.073	0.051	0.000
	0.912	0.912	0.072	0.079	0.000
	0.915	0.915	0.073	0.079	0.000
	0.884	0.884	0.071	0.073	0.000
	0.904	0.904	0.073	0.073	0.000
	0.898	0.898	0.071	0.080	0.000
	0.871	0.871	0.070	0.073	0.000
	0.899	0.899	0.073	0.087	0.000
	0.893	0.893	0.073	0.087	0.000
Rockall	0.994	0.994	0.718	0.430	0.136
	0.997	0.997	0.715	0.523	0.154
	0.990	0.990	0.299	0.442	0.199

DBEM CMIP3-E	AquaMaps GFDL	AquaMaps CMIP3-E	Maxent GFDL	Maxent CMIP3-E	DBEM GFDL	DBEM CMIP3-E
	Difference in habitat suitability (2050-1985)					
0.040	0.000	0.000	-0.014	0.027	0.000	0.003
0.034	0.000	0.000	-0.023	0.022	0.000	0.002
0.035	0.000	0.000	-0.022	0.002	0.000	0.003
0.029	0.000	0.000	-0.027	0.002	0.000	-0.029
0.030	0.000	0.000	-0.005	0.027	0.000	-0.030
0.033	0.000	0.000	-0.023	0.021	0.000	0.002
0.044	0.000	0.000	-0.058	-0.017	0.000	0.003
0.025	0.000	0.000	-0.076	-0.017	0.000	-0.025
0.000	0.000	0.000	-0.078	-0.023	0.000	0.000
0.022	0.000	0.000	-0.044	-0.022	0.000	-0.022
0.030	0.000	0.000	-0.032	-0.012	0.000	0.002
0.057	0.000	0.000	-0.077	-0.018	0.000	0.004
0.039	0.000	0.000	-0.015	-0.021	0.000	0.002
0.037	0.000	0.000	-0.025	-0.022	0.000	0.002
0.000	0.000	0.000	-0.080	-0.031	0.000	0.050
0.000	0.000	0.000	-0.083	-0.030	0.000	0.036
0.000	0.000	0.000	-0.085	-0.031	0.000	0.039
0.000	0.000	0.000	-0.089	-0.054	0.000	0.047
0.000	0.000	0.000	-0.005	-0.008	0.000	0.000
0.000	0.000	0.000	-0.005	-0.008	0.000	0.000
0.000	0.000	0.000	-0.002	0.000	0.000	0.000
0.000	0.000	0.000	-0.003	0.000	0.000	0.000
0.000	0.000	0.000	-0.003	-0.009	0.000	0.000
0.000	0.000	0.000	-0.003	-0.009	0.000	0.000
0.000	0.000	0.000	0.000	-0.008	0.000	0.000
0.000	0.000	0.000	0.000	-0.009	0.000	0.000
0.000	0.000	0.000	-0.001	-0.001	0.000	0.000
0.000	0.000	0.000	0.000	-0.008	0.000	0.000
0.000	0.000	0.000	0.000	-0.001	0.000	0.000
0.000	0.000	0.000	0.000	-0.001	0.000	0.000
0.163	0.000	0.000	-0.031	-0.042	0.019	0.048
0.183	0.000	0.000	-0.026	-0.073	0.021	0.051
0.237	0.000	0.000	-0.023	-0.034	0.027	0.066
